# Weaning failure due to isolated residual diaphragmatic paralysis after cervical spinal cord ischemia following aortic surgery- a case report

**DOI:** 10.1186/s12871-024-02626-2

**Published:** 2024-07-17

**Authors:** Remco Overbeek, Amelie Behrens, David Zopfs, Spyridon Mylonas, Bernhard Dorweiler, Fabian Dusse, Bernd W. Böttiger, Sandra Emily Stoll

**Affiliations:** 1 Department of Anesthesiology and Intensive Care Medicine, Faculty of Medicine and University Hospital Cologne, University of Cologne, 50937 Cologne, Germany; 2 Department of Vascular and Endovascular Surgery, Faculty of Medicine and University Hospital Cologne, University of Cologne, 50937 Cologne, Germany; 3 Faculty of Medicine and University Hospital Cologne, Department of Diagnostic and Interventional Radiology, University Cologne, 50937 Cologne, Germany

**Keywords:** Weaning failure, Spinal ischemia, Diaphragmatic paralysis

## Abstract

**Background:**

Bilateral diaphragmatic dysfunction can lead to dyspnea and recurrent respiratory failure. In rare cases, it may result from high cervical spinal cord ischemia (SCI) due to anterior spinal artery syndrome (ASAS). We present a case of a patient experiencing persistent isolated diaphragmatic paralysis after SCI at level C3/C4 following thoracic endovascular aortic repair (TEVAR) for Kommerell’s diverticulum. This is, to our knowledge, the first documented instance of a patient fully recovering from tetraplegia due to SCI while still exhibiting ongoing bilateral diaphragmatic paralysis.

**Case presentation:**

The patient, a 67-year-old male, presented to the Vascular Surgery Department for surgical treatment of symptomatic Kommerell’s diverticulum in an aberrant right subclavian artery. After successful surgery in two stages, the patient presented with respiratory insufficiency and flaccid tetraparesis consistent with anterior spinal artery syndrome with maintained sensibility of all extremities. A computerized tomography scan (CT) revealed a high-grade origin stenosis of the left vertebral artery, which was treated by angioplasty and balloon-expandable stenting. Consecutively, the tetraparesis immediately resolved, but weaning remained unsuccessful requiring tracheostomy. Abdominal ultrasound revealed a residual bilateral diaphragmatic paralysis. A repeated magnetic resonance imaging (MRI) 14 days after vertebral artery angioplasty confirmed SCI at level C3/C4. The patient was transferred to a pulmonary clinic with weaning center for further recovery.

**Conclusions:**

This novel case highlights the need to consider diaphragmatic paralysis due to SCI as a cause of respiratory failure in patients following aortic surgery. Diaphragmatic paralysis may remain as an isolated residual in these patients.

**Supplementary Information:**

The online version contains supplementary material available at 10.1186/s12871-024-02626-2.

## Introduction

Bilateral diaphragmatic dysfunction is a known, yet underdiagnosed cause of dyspnea and recurrent respiratory failure. It is usually caused by chest or neck surgery, neck injury, manipulation of the cervical spine, neuromuscular diseases or cardiac surgery [1]. In some instances, diaphragmatic paralysis may be caused by high cervical spinal cord ischemia (SCI) due to anterior spinal artery syndrome (ASAS). The anterior spinal artery runs ventrally along the entire length of the spinal cord and supplies its anterior two-thirds [2]. Ischemic infarction due to direct occlusion or hypoperfusion of feeding arteries can lead to ASAS [3]. Only a few cases have been described where diaphragmatic paralysis persisted, while tetraplegia receded after SCI [4]. This is, to our knowledge, the first patient fully recovering from tetraplegia due to SCI continuing to present bilateral diaphragmatic paralysis resulting in weaning failure from mechanical ventilation.

## Case report

**Fig. 1:**
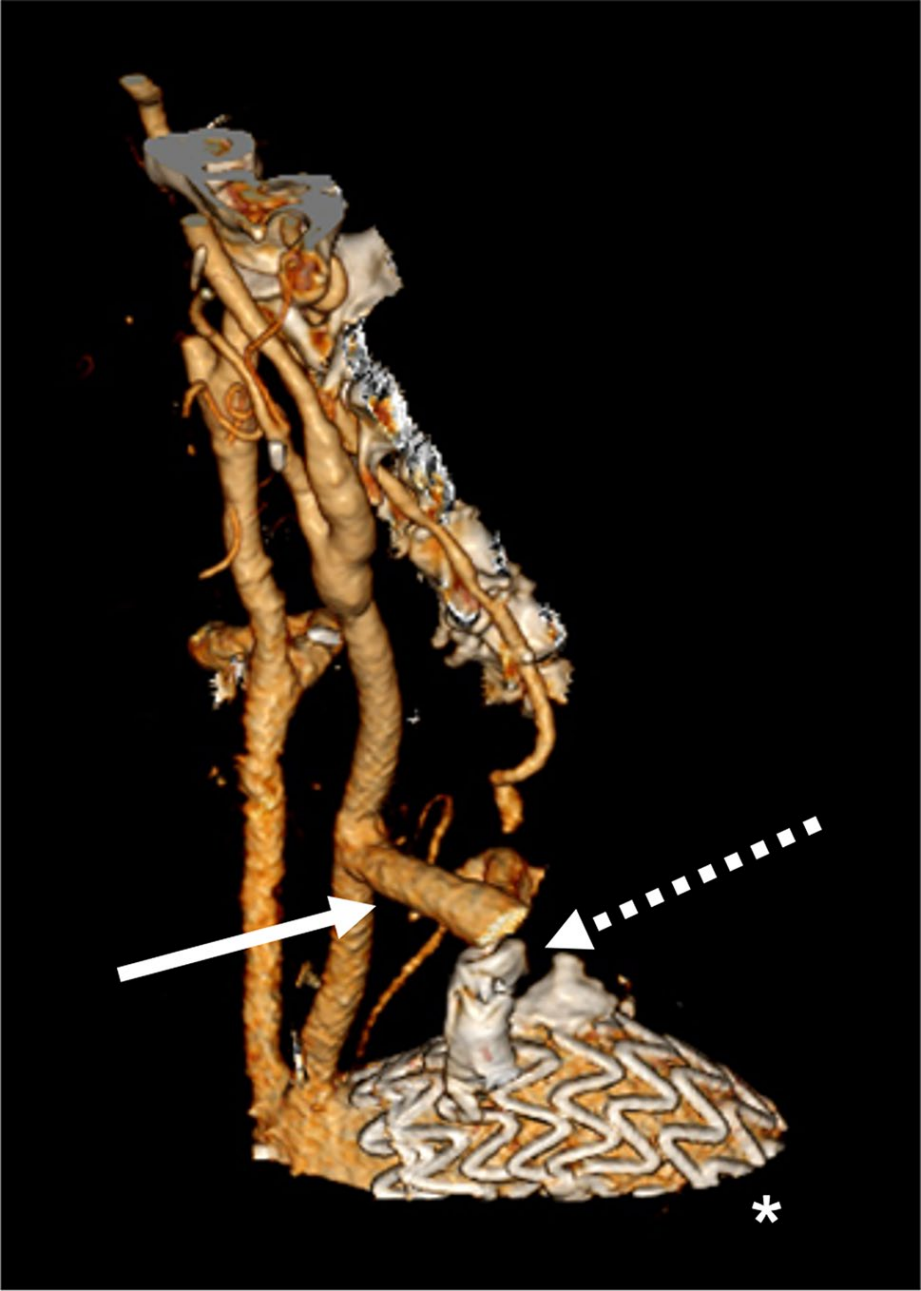
3D reconstruction of the postoperative CT (computed tomography) angiography. The thoracic stent prosthesis (*), the vascular plug implanted in the left proximal subclavian artery (dashed arrow) and the bypass between the common carotid artery and the subclavian artery (arrow) are shown. In addition, the high-grade stenosis of the left vertebral artery can be seen

A 67-year-old male caucasian patient presented to the Vascular Surgery Department for surgical treatment of symptomatic Kommerell’s diverticulum in an aberrant right subclavian artery. Progressive hoerseness was described for at least the last 6 months combined with upper thoracic pain the last two weeks The patient had a history of endarterectomy of the left and right carotid artery (lefts side 5 months and right side 18 years before), respectively, for high-grade asymptomatic carotid stenosis in another hospital. In addition, the patient had suffered from a previous stroke (9 years before). Due to the complexity of the anatomy and after intedersciplinary case discussion in our aortic board (cardiac surgeons, vascular surgeons, anesthesiologists, cardiologists) an urgent hybrid approach in tho stages was chosen. Informed consent was obtained from the patient and a transposition of the right vertebral and subclavian arteries to the right common carotid artery was undertaken. The patient had an uneventful postoperative course and three days later, the second step with a left-sided carotid-subclavian bypass combined with an endovascular exclusion (implantation of a thoracic aortic stentgraft and a vascular plug in the proximal left subclavian artery) of the Kommerell’s diverticulum was successfully performed. In the immediate postoperative course the patient could be extubated without neurological deficits. On the second postoperative day the patient developed a complete flaccid tetraplegia with reduced reflexes in the left arm and increased reflexes in the remaining limbs. Additionally, the patient developed progressive respiratory failure requiring reintubation. The immediately performed computed tomographyangiography (CTA) detected a flow-limiting dissection of the proximal right vertebral artery and a high-grade origin stenosis of the left vertebral artery (Fig. 1).

**Fig. 2:**
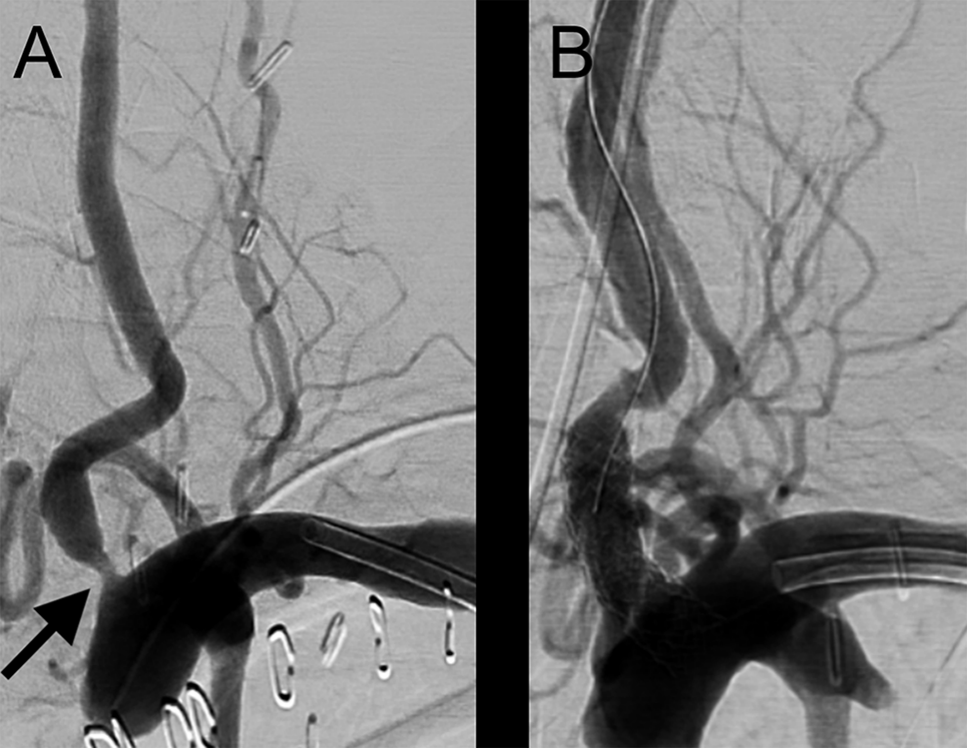
Transbrachial digital subtraction angiography of the left subclavian artery after retrograde distal brachial artery access. A high-grade stenosis of the vertebral artery near the origin (A, left side) is shown (arrow). The stenosis was treated by percutaneous trans-luminal angioplasty (balloon-PTA and stenting), resulting in a significant reduction of the stenosis (B, right side)

After consultation of the neurologists and neuroradiologists the suspicion of cervical spinal cord ischemia as cause of tetraparesis was raised and an angioplasty with stenting of the left vertebral artery was conducted (Fig. 2).

**Fig. 3:**
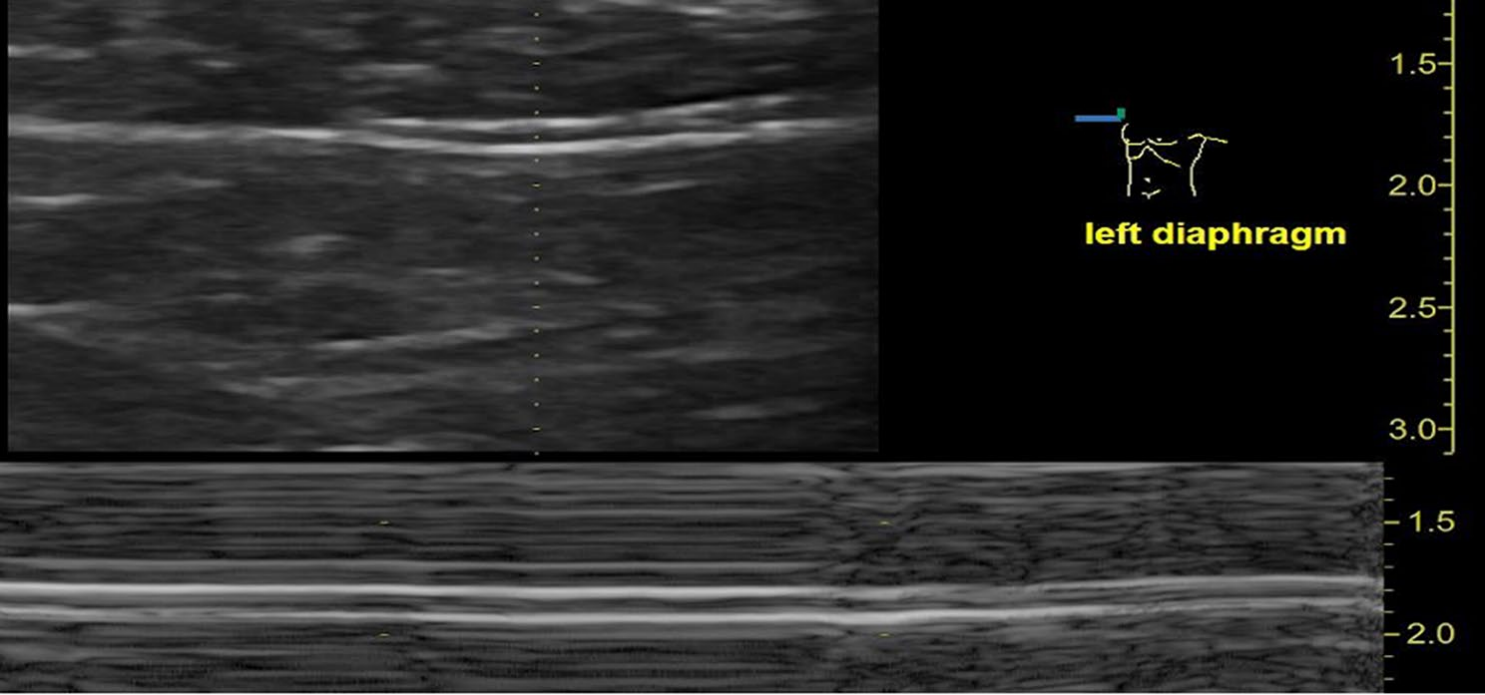
Ultrasound of the left diaphragm: No diaphragmatic thickening and elevation was detected

The tetraplegia was fully reversible thereafter, but the patient continued to present with bilateral diaphragmatic paralysis (absent diaphragmatic thickening / diaphragmatic elevation in abdominal ultrasound) (Fig. 3, Video of ultrasound of left and right diaphragm).

**Fig. 4:**
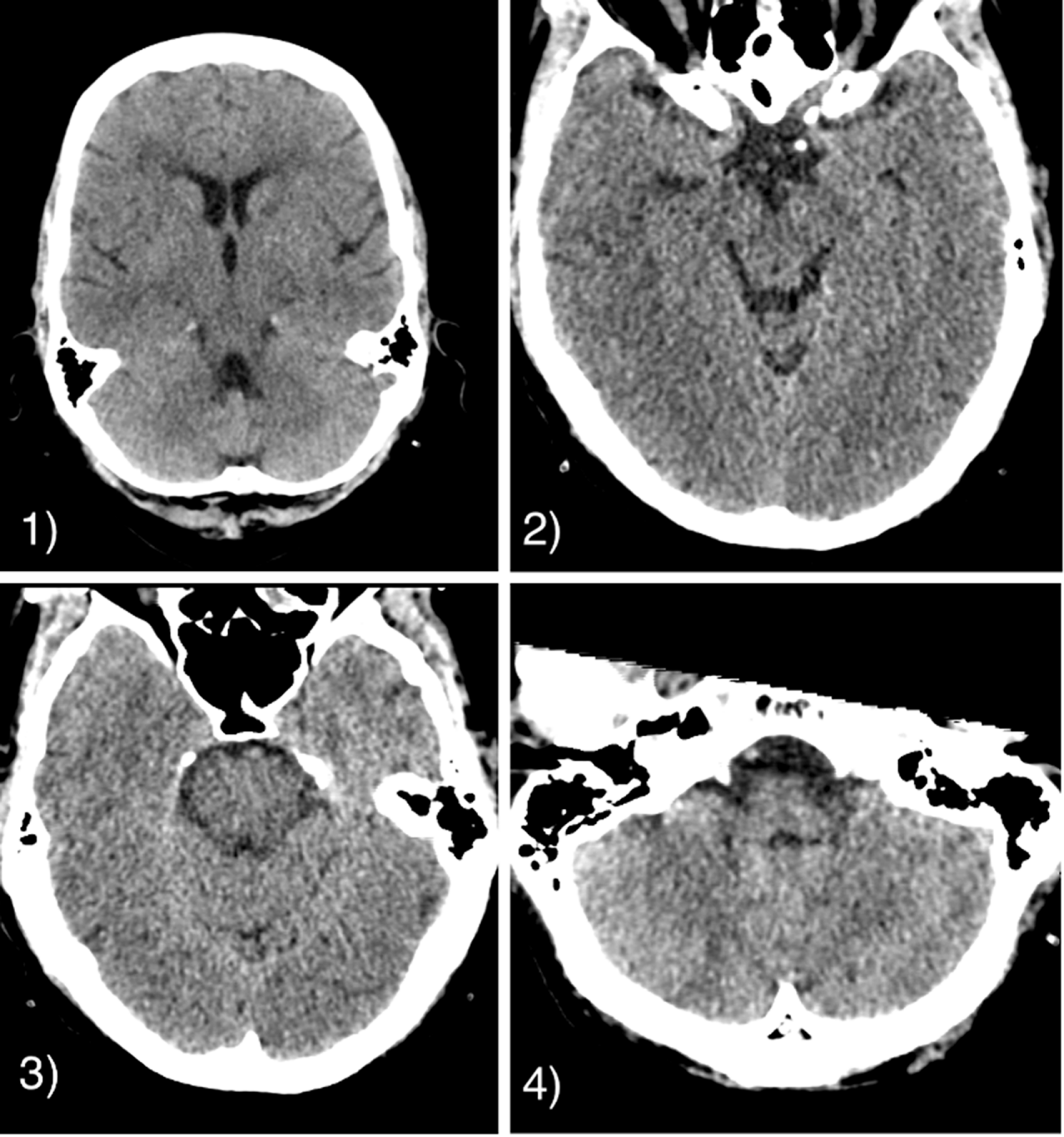
Unremarkable axial CT images one day prior to angiographic treatment of the vertebral artery stenosis. Levels: (1) brain stem (5 mm slice thickness), (2) mesencpehalon, (3) pons, (4) medulla oblongata (all 1 mm slice thickness)

**Fig. 5:**
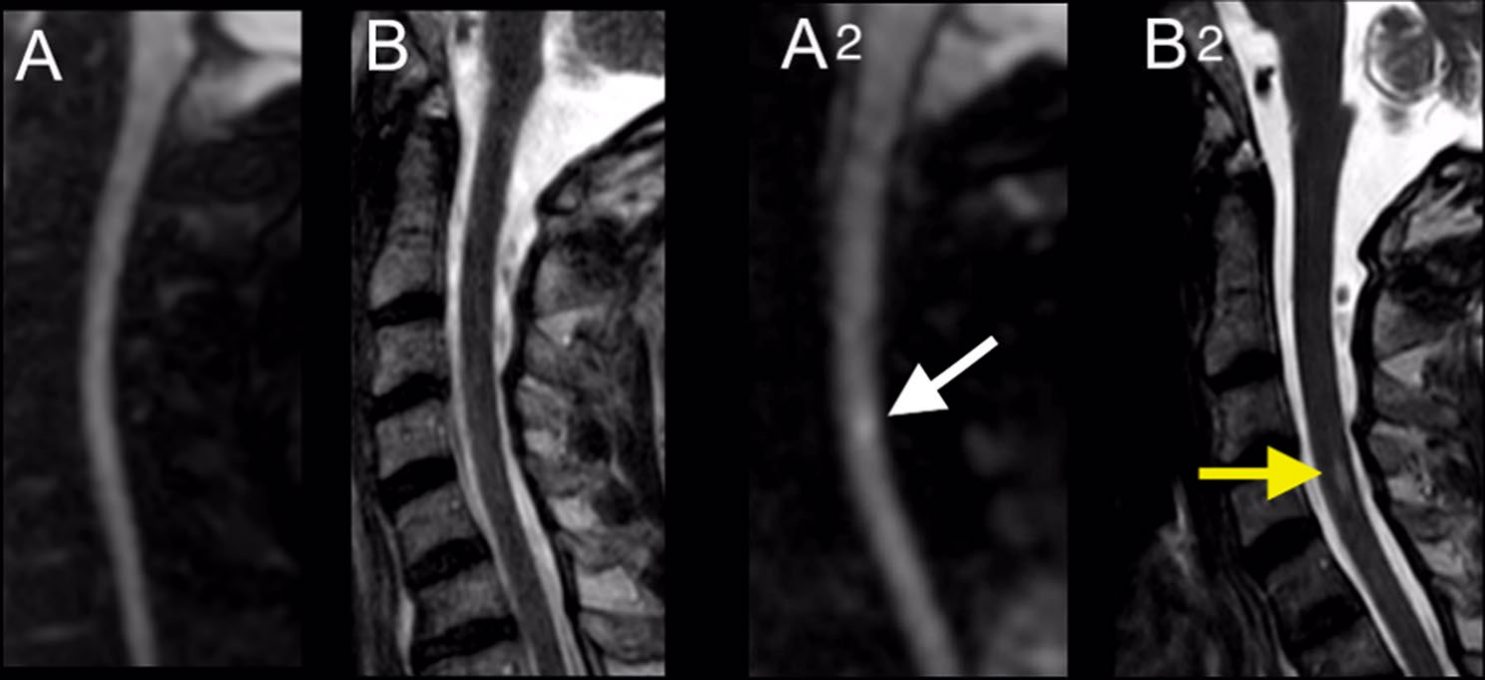
Left: MRI of the cervical spine in sagittal slice orientation. Diffusion-weighted sequence (**A**), and T2 sequence (**B**) show no evidence of spinal ischemia. Right: Follow-Up MRI of the cervical spine in sagittal slice orientation. A dot-shaped diffusion restriction (white arrow) is seen in the diffusion-weighted sequences (A2) with a hyperintense correlate (yellow arrow) in the T2 sequence (B2) at the level of the fourth cervical vertebral body, consistent with spinal ischemia

Subsequently, weaning attempts remained futile, leading to the decision to perform a tracheostomy. The initial magnetic resonance imaging (MRI) scan one day after vertebral artery angioplasty showed no signs of SCI. A cerebral CT did not show any signs of infarction or bleeding (Fig. 4). However, a follow-up MRI scan after 2 weeks showed findings consistent with segmental SCI at level C3/C4 (Fig. 5).

The patient was eventually transferred to a pulmonary clinic with a specialised weaning center for further recovery. Three months after the patient’s discharge, bilateral diaphragmatic paralysis remained unchanged.

## Discussion and conclusions

Due to the rarity and heterogeneity of high cervical SCI, to date definite treatment options are lacking and therapeutic recommendations are mostly based on supportive measures to increase arterial perfusion of the spinal cord [5, 6]. Medical management options of spinal cord ischemia include treatment with thrombolysis and early corticosteroid administration for improved functional recovery, though it remains unclear whether this advantage extends to individuals with vascular cord ischemia [7, 8]. When the patient initially presented with tetraplegia, the differential diagnosis for SCI included Guillain-Barré syndrome, hypokalemic periodic paralysis, and ischemic stroke of the posterior circulation. All of the least diagnoses could be ruled out via cerebral angiogram/CT scan, normal potassium levels and the clinical picture and progress of the case. A cerebral MRI might have been beneficial in ruling out cerebral infarction and could have been applied parallel to cervical MRI to detect SCI [9], but the second pathological cervical MRI scan fully explained the acute onset and the clinical picture of SCI.

In this case the spinal perfusion was impaired by the high-grade stenosis of the vertebral artery. The decision to perform balloon dilatation and stent implantation was made due to the severity of symptoms, the acute onset and the vertebral artery stenosis as a treatable cause. Clinical trials have highlighted the safety and efficacy of endovascular therapy for treating vertebral artery stenosis, though there is still limited evidence demonstrating the superiority of endovascular treatment over the best medical management [10, 11].

A high SCI affecting level C3 to C5 can cause damage to the phrenic nerves leading to diaphragmatic paralysis, as presented in our case in the form of weaning failure from the ventilator [12]. While diaphragmatic dysfunction is a frequent complication observed in cardiac surgery, primarily caused by direct phrenic nerve injury resulting from hypothermia or mechanical trauma [3], it is very uncommon to result from SCI. In this case, suspicion of SCI arose when the patient developed tetraparesis consistent with symptoms of ASAS. Since the patient failed to be weaned from the ventilator and presented an abnormal breathing pattern mainly using his auxiliary respiratory and abdominal muscles for ventilation, an abdominal ultrasound was performed. This finally unveiled bilateral diapgragmatic paralysis without diaphragmatic elevation. At this point, the tetraplegia had fully resolved and the patient presented neurologically unremarkable, leaving the diaphragmatic paralysis as the only residual damage. To our knowledge, comparable cases have not yet been described in literature.

Diagnosing phrenic nerve paralysis can be challenging, especially when the pathogenesis is as complex as in this case. On suspicion of phrenic nerve paralysis, diagnostic procedures in addition to patients past medical history should include physical examination, blood gas analysis, lung function testing and diagnosis of the underlying disease. This should be followed by specific respiratory muscle testing and respiratory imaging such as diaphragmatic ultrasound [13]. In this case, paralysis was diagnosed by abdominal ultrasound, revealing a flat, non-thickening, non-contracting diaphragm bilaterally. Abdominal ultrasound is an easy, non-invasive and cost-efficient way to assess and reevaluate diaphragmatic function [14].

Due to the patient’s clinical presentation, prolonged weaning was expected, and early tracheostomy was performed which has been associated with shorter duration of mechanical ventilation, shorter length of ICU stay and fewer laryngotracheal complications in patients with traumatic SCI [15]. Future therapeutic options for the patient could include inspiratory respiratory muscle training [16] and intrathoracic phrenic pacing [17]. Current expert opinion recommends reassessing recovery of the phrenic nerve and diaphragm after a time- period of 3 to 6 months after the initial injury. The need for a pacer for phrenic nerve stimulation should be reevaluated after this period. [18].

Our case demonstrates that weaning failure after aortic surgery may be caused by diaphragmatic paralysis due to cervical SCI. Of note, diaphragmatic paralysis may remain as an isolated residual in these patients.

### Electronic supplementary material

Below is the link to the electronic supplementary material.

Supplementary Material 1

Supplementary Material 2

## Data Availability

The datasets analysed during the current study are available from the corresponding author on reasonable request.

## Abbreviations

SCI: Spinal Cord Ischemia
TEVAR: Thoracic Endovascular Aortic Repair
CT: Computerized Tomography Scan
MRI: Magnetic Resonance Imaging
ASAS: Anterior Spinal Artery Syndrome
