# Supplementary figures and images for: Weaning failure due to isolated residual diaphragmatic paralysis after cervical spinal cord ischemia following aortic surgery- a case report
Source: BMC Anesthesiol. 2024 Jul 17;24:241. doi: 10.1186/s12871-024-02626-2 (PMC11253376; doi:10.1186/s12871-024-02626-2)

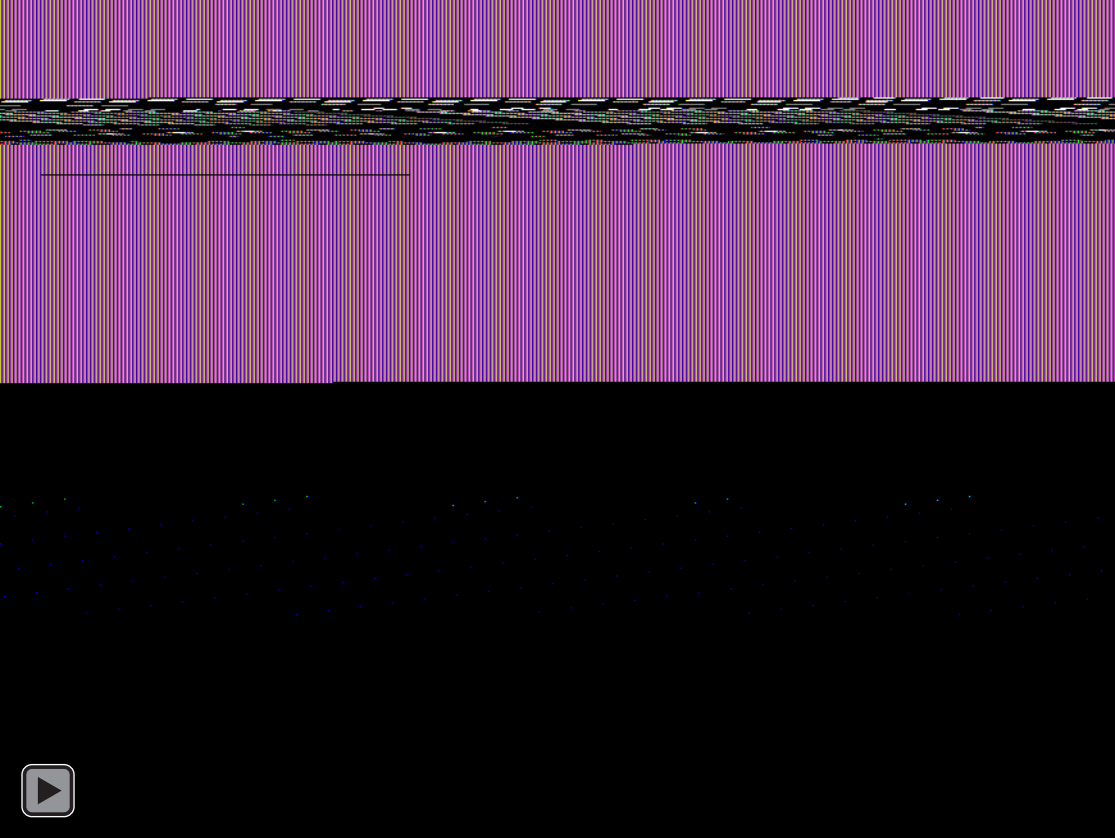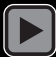

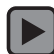

Supplement: Supplementary file 1 — Supplementary Material 1 [file 12871_2024_2626_MOESM1_ESM.pdf]
