# Supplementary material for: Weaning failure due to isolated residual diaphragmatic paralysis after cervical spinal cord ischemia following aortic surgery- a case report
Source: BMC Anesthesiol. 2024 Jul 17;24:241. doi: 10.1186/s12871-024-02626-2 (PMC11253376; doi:10.1186/s12871-024-02626-2)
Supplement: Supplementary file 2 — Supplementary Material 2 [file 12871_2024_2626_MOESM2_ESM.doc]

Supplementary videos 1 + 2

Ultrasound of left (1) and right (2) diaphragm revealing a flat, non-thickening, non-contracting diaphragm bilaterally.
